# *Isolation*, identification, biological characteristics analysis, and inactivated vaccine research of *Mycoplasma canis* from respiratory tract of dogs in Yunnan Province, China

**DOI:** 10.3389/fvets.2025.1716004

**Published:** 2025-12-24

**Authors:** Meiling Kou, Tao Xu, Yinan Wang, Jiarui Xie, Jiangyan Huang, Yun Xin, Chao Chen, Qing Fan, Aiguo Mao, Haisheng Miao

**Affiliations:** 1 Yunnan Tropical and Subtropical Animal Virus Disease Laboratory, Yunnan Academy of Animal Husbandry and Veterinary Sciences, Kunming, Yunnan, China; 2 Kunming Police Dog Base of the Ministry of Public Security, Kunming, Yunnan, China; 3 Department of Biotechnology, Guangxi Veterinary Research Institute, Nanning, Guangxi, China

**Keywords:** *Mycoplasma canis*, isolation and identification, 16S rRNA analysis, biological characteristics, inactivated vaccine

## Abstract

**Introduction:**

Research on *Mycoplasma canis* remains limited, possibly because dogs in most regions are usually not raised on a large scale or in intensive farming. Furthermore, existing antibacterial drugs are often proven to be effective in inhibiting or eradicating *M. canis*, resulting in insufficient attention being paid to its pathogenic potential. However, with the widespread use of antibacterial drugs, an increase in drug resistance has been found in human *mycoplasma pneumoniae*, bovine mycoplasma and avian mycoplasma also faces similar challenges. Given the irreplaceable role of dogs as companion animals and work partners, it is crucial to enhance research on Mycoplasma canine, especially in the areas of epidemiology, diagnostic techniques, drug resistance mechanisms and vaccine development. This study found that *M. canis* inoculated into liquid Pleuropneumonia-Like Organisms (PPLO) medium at 37 °C could reach the growth peak within 24 h and maintain this level for approximately 24 h. The survival rate is higher in environments with PH values of 6.5, 7.0 and 7.5 respectively, and it is suitable for storage in neutral or weakly acidic environments. 5 mM of BEI inactivated agent can completely inactivate *M. canis* within 15 h, and the experimental dogs produced specific antibodies within 1 week after vaccination with the inactivated vaccine, with a positive rate of 100%. It provides a theoretical basis and experimental evidence for the subsequent in-depth exploration of the pathogenic mechanism of *M. canis* and the development of its vaccine.

**Methods:**

Researchers collected eight lung tissue samples from working dogs exhibiting typical respiratory symptoms in Kunming City, Yunnan Province. From these samples, one strain of canine mycoplasma was successfully isolated. To characterize this isolate comprehensively, a series of technical approaches were employed. The growth dynamics of the mycoplasma strain were assessed by monitoring color changes in the culture medium. Molecular identification was conducted using polymerase chain reaction (PCR). Additionally, preliminary studies were undertaken regarding its biological characteristics and the development of an inactivated vaccine; these efforts provide both theoretical foundations and experimental support for future investigations into the pathogenic mechanisms and vaccine development for *M. canis.*

**Results:**

The findings indicated that *M. canis* could grow stably within PPLO medium and was confirmed as belonging to the *M. canis* genotype through 16S rRNA gene sequence analysis. The freshly cultivated strain was inoculated into liquid PPLO medium at a concentration rate of 1%. It reached its peak growth within 24 h at 37 °C and maintained this level for approximately another 24 h. Subsequently, the number of viable bacteria gradually declined. In environments with pH values of 6.5, 7.0, and 7.5 respectively, the survival rate of viable bacteria was significantly higher than that observed at pH 8.0. Therefore, it is recommended that the *M. canis* strain preserved for seed use be maintained in a neutral or weakly acidic environment. Additionally, the BEI inactivating agent at a final concentration of 5 mM can completely inactivate the strain within 15 h. Four experimental dogs developed specific antibodies within 1 week after being vaccinated with the inactivated vaccine, achieving a positive rate of 100%, which preliminarily confirms that the vaccine elicits an immune response.

**Conclusion:**

This study successfully isolated and identified *M. canis*, investigated its growth characteristics in PPLO medium, and established a method for preparing inactivated vaccines along with preliminary evaluations thereof. These findings provide a foundation for further research into the infection dynamics and pathogenicity of *M. canis* while offering valuable insights for preventing and controlling *M. canis* infections.

## 1 Introduction

Mycoplasma represents some of the smallest known prokaryotic organisms, measuring between 0.3 to 0.8 micrometers in diameter. Its unique lack of a cell wall positions it between bacteria and viruses regarding structural characteristics. These microorganisms rely on host-derived cholesterol, sterols, or urea as essential growth factors and are commonly found colonizing mammalian respiratory mucosa (1, 2). It is noteworthy that expanding host ranges lead to ambiguity surrounding *Mycoplasma canis* concepts since identical species may be isolated from multiple hosts (1).

The first documented occurrence of mycoplasmas in dogs was in 1934. To date, 15 clearly classified species of canine mycoplasma have been isolated and identified, with an additional two species yet to be fully classified or named (1, 2). Notably, the expansion of host range has led to ambiguity in the definition of canine mycoplasma, as the same species may be isolated from multiple host species (1). The genetic diversity of canine mycoplasma has not been fully elucidate, and potential genetic variations may affect its pathogenic mechanism (1). Due to the lack of standardized diagnostic methods for canine mycoplasma at present, it is difficult to distinguish contaminated samples from infected ones (3). Subsequent research combines metagenomics and functional genomics to explore the molecular mechanism of canine mycoplasma virulence factors and establish a host-pathogen interaction model. A study has indicated that in dogs with lower respiratory tract disease (LRT), although *mycoplasma* was detected, some researchers suggest that it may reflect oropharyngeal contamination rather than direct pathogenicity (3, 4). However, canine mycoplasma has been isolated from dogs with pneumonia and is particularly prevalent in the most necrotic regions of the lungs (5). Moreover, in a case of severe bronchopneumonia affecting a litter of puppies, canine mycoplasma was the sole pathogen identified. This infection resulted in the death of some puppies, while the surviving ones recovered following appropriate antibiotic treatment (6).

In recent years, an outbreak of respiratory diseases has occurred in a large-scale working dog breeding base in Kunming City, Yunnan Province. The infected dogs generally showed typical respiratory clinical symptoms such as coughing and breathing difficulties, and there have been reports of deaths. Pathological examination revealed that the lungs of the affected dog presented obvious hemorrhagic lesions, tissue degeneration and purulent inflammation. Pathological examination revealed that the lungs of the affected dog presented obvious hemorrhagic lesions, tissue degeneration and purulent inflammation. According to statistics, According to statistics, there were 963 cases of respiratory tract infectioned in 2023 and this number had been increased to 1,313 in 2024. During the 2 years, there were a total of five concentrated outbreaks of respiratory diseases. Most dogs mainly present with subclinical or mild symptoms of the upper respiratory tract such as dry cough or wet cough. The symptoms become more obvious during exercise, excitement or sudden changes in temperature. The course of the disease usually lasts for 1 to 2 weeks. When some dogs have been ill for more than 2 weeks, they often show systemic symptoms such as fever, loss of appetite and depression. In some cases, breathing difficulties may even occur, presenting as abdominal breathing. In the middle and later stages of the disease, secondary infections are relatively common, which in turn can lead to lobar pneumonia. During the most recent epidemic, 22 cases were tested by mycoplasma fluorescence quantitative PCR. The results showed that canine mycoplasma nucleic acid was positive in upper respiratory tract or lung tissue samples. At the same time, Bordetella was not detected, but some cases were positive for canine parainfluenza virus nucleic acid. In this study, the lungs of infected dogs were collected for *mycoplasma* isolation and identification. *M. canis* strains that could be stably subcultured on PPLO liquid medium continuously were obtained. The 16S rRNA gene, growth characteristics, and stability of these strains were studied and analyzed. The immune effect of inactivated vaccines was preliminarily prepared and evaluated. It is expected to provide theoretical and technical support for the prevention and control of diseases caused by *M. canis* infection.

## 2 Materials and methods

### 2.1 Collection of diseased materials and isolation of *M. canis*

Euthanasia was performed on Kunming dog breeds exhibiting terminal conditions due to Mycoplasma infection at the Kunming Working Dog Breeding Base. Preoperative sedation was induced via intravenous administration of propofol at a dosage of 0.5 mL/kg to achieve rapid sedation and muscle relaxation, thereby minimizing stress or distress associated with physical restraint. Following complete sedation, a lethal dose of potassium chloride (0.5 mL/kg) was administered intravenously to induce cardiac arrest. The entire euthanasia procedure lasted from several seconds to tens of seconds, during which the animals experienced no pain or discomfort. Postmortem lung specimens were collected from the deceased dogs at Anle for subsequent pathological analysis. Following euthanasia, lungs were dissected and harvested. Tissue samples were obtained from multiple lesion sites, sectioned into small pieces, and homogenized in an appropriate volume of PPLO medium. The homogenate was centrifuged at 500 × g for 10 min, and the supernatant was filtered through a 0.45 μm membrane filter. The filtrate was inoculated into PPLO liquid medium at a ratio of 1:10 and incubated at 37 °C. The PPLO liquid medium was prepared as follows: 21 g of PPLO broth base, 2 g of glucose, 5 g of yeast extract, 2 g of sodium pyruvate, and 1% phenol red were dissolved in 600 mL of ultrapure water with stirring. The pH was adjusted to 7.6–7.8 using 1 M sodium hydroxide solution, and the volume was brought to 800 mL. The medium was sterilized by autoclaving at 121 °C for 15 min. After cooling to approximately 40 °C, 20% horse serum and 100,000 units of penicillin were added aseptically. The prepared medium was stored at 4 °C until use. After the initial culture period of 3 days, the culture medium was retrieved and filtered again through a 0.45 μm membrane filter. A 1:10 volume of the filtrate was then transferred into fresh PPLO medium for subculture. Culture completion was determined based on color change of the medium; vigorous Mycoplasma growth resulted in a shift from red to yellow, indicating acidification due to metabolic activity.

### 2.2 Analysis of 16S ribosomal RNA gene sequence

Mycoplasma isolated and cultured to the second generation on PPLO medium was designated YNKM2024. Following nucleic acid extraction, 16S ribosomal RNA gene (16S rRNA) amplification was performed using the PrimeScript TaKaRa One Step RT-PCR Kit Ver.2, and the resulting PCR products were sequenced. The 16S rRNA gene fragment was amplified in two overlapping segments via PCR. The primer sequences and corresponding reaction conditions are summarized in Table 1. After sequencing, the obtained gene fragments were assembled using SeqMan software. Reference sequences were retrieved from the NCBI database for sequence alignment and phylogenetic analysis (Table 2). A positive control plasmid containing the 16S rRNA gene of *M. canis* was synthesized based on the *M. canis* reference sequence available in NCBI.

**Table 1:** Primers and reaction conditions.

Gene	Primer Sequences	Tm	Length of product
*Mycoplasma canis*	Seg-1-F1-AGAGTTTGATCCTGGCTCAGGATGA	55.0 °C	728 bp
	Seg-1-R1-AGTTAGCTGCCTTCGCCATGTTG		
*M. canis*	Seg-2-F2-TAGAGGTTAGCGGAATTCCTAGT	55.0 °C	835 bp
	Seg-2-R2-GTTCTCGTAGGGATACCTTGTTA		

**Table 2:** Names and sources of reference genes.

No.	GenBank accession number	Country	Year of collection	Part or variety	Gene
1	LR215012	UK	1951	Throat of canine	*M. canis*
2	FJ666136	USA	/	/	*M. canis*
3	CP011368	USA: Florida	1992	*Canis lupus familiaris* breed German Shepherd	*M. canis*
4	MK615079	Austria	2018	*Vulpes vulpes* (Red fox)	Mycoplasma sp.
5	FJ876261	USA	/	/	*M. canis*
6	PV262276	Iraq: Wasit	2025	Nasal swab of canine	*M. canis*
7	LS991951	Wellcome Trust Sanger Institute	1951	Throat of Dog	*Mycoplasma edwardii*
8	MN280655	Mexico	/	Blood of *Canis lupus familiaris*	*M. canis*
9	PV662303	Iraq: Wasit	2025	Nasal swab of dog	*M. cynos*
10	PV262271	Iraq: Wasit	2025	Nasal swab of canine	*M. canis*
11	CP141046	Canada: Guelph, Ontario	2018	Bronchoalveolar lavage of *Canis lupus familiaris*	*M. cynos*
12	AF340023	Norway	2001	Urinary sediment of dog	*M. canis* PG14
13	U73903	USA	1996	/	*M. edwardii* PG24
14	AF538682	UK	2002	/	*M. cynos* H381
15	U09787	USA	1994	Felis domesticus	*M. felis*

### 2.3 Growth curve of Mycoplasma in the canine respiratory tract

The fifth-generation mycoplasma strain, freshly cultured, was thoroughly resuspended and aliquoted into 1 mL volumes per tube, followed by storage at −80 °C. One aliquot was retrieved for color change unit (CCU) titration. For CCU determination, the sample was subjected to serial 10-fold dilutions in PPLO liquid medium using microcentrifuge (EP) tubes, with dilution factors ranging from 10^−1^ to 10^−12^. Following inoculation, the diluted samples were incubated at 37 °C for 7 days, after which results were interpreted: tubes exhibiting mycoplasma growth were indicated by a color shift to yellow, whereas negative controls retained a red color. The CCU titer was defined as the highest dilution showing positive growth. The initial CCU titer of this batch was determined to be 10^8^. An additional aliquot from the same batch was inoculated into 10 mL of PPLO liquid medium at a 1% (v/v) concentration and incubated at 37 °C under static conditions. Aliquots of 500 μL were collected at predetermined time points—0, 6, 12, 18, 24, 30, 36, 42, 48, 56, 60, 66, and 72 h post-inoculation—for immediate CCU analysis. Each time point was assayed in triplicate. Samples were serially diluted (10-fold steps) in PPLO medium across the same dilution range (10^−1^ to 10^−12^), incubated at 37 °C for 5 days, and assessed for color change, with yellow indicating mycoplasma proliferation.

### 2.4 The influence of pH value on the stability of *M. canis*

Mycoplasma canine was cultured using the above-mentioned PPLO medium. When the pH value turned to 6.5, the mycoplasma titer was detected by the CCU method. At the same time, the mycoplasma was divided into 4 portions. The pH values were adjusted to 6.5, 7.0, 7.5, and 8.0, respectively, with sodium hydroxide solution and placed at 4 °C. Thereafter, samples of each different pH value were taken for CCU detection every 2 days. The results of each sample tested were read at 5 days to evaluate the stability of *M. canis* under the storage condition of 4 °C.

### 2.5 Preparation of inactivated vaccines and immunization experiments

500 mL of *M. canis* was cultured in PPLO liquid medium. The culture was completed when the pH value of the medium was 6.5 and the liquid turned yellow. Place the culture in a Beckman SW32 rotor centrifuge tube and centrifuge at 12,000 rpm/min for 30 min. Discard the supernatant, resuspend the precipitate with 0.02 M PBS buffer, using a total of 50 mL of PBS buffer. Store in a − 80 °C refrigerator for future use. The bacterial liquid was inactivated with BEI inactivator at a final concentration of 5 mM at 37 °C. During the inactivation process, a magnetic rod was used for gentle stirring, and the inactivation container was replaced after 10 h of inactivation. After inactivation for 15 h, sodium thiosulfate solution with a final concentration of 10 mM was added to terminate the inactivation, and samples were taken and inoculated into PPLO medium to verify safety. The Montanide Del 02 PR adjuvant from Seppic of France was added to the inactivated mycoplasma suspension at a rate of 15% (v/v), thoroughly mixed to prepare the inactivated vaccine. Six dogs with negative mycoplasma antibodies and oral and nasal swab antigens were selected as experimental dogs at the Kunming Police Dog Base. Vaccination experiments were carried out in the isolation kennel. Among them, four dogs were vaccinated with 1 mL of inactivated vaccine each, and two dogs were used as negative controls and vaccinated with the same emulsified PBS. Serum samples were collected at 0,1,2, and 3 weeks after vaccination for mycoplasma antibody detection. Evaluate the immune effect of the vaccine. The detection method for *M. canis* antibodies is the cELISA antibody detection method for *M. canis* prepared by the Yunnan Academy of Animal Husbandry and Veterinary Sciences.

## 3 Discussion

### 3.1 Mycoplasma isolation

**Figure 1:**
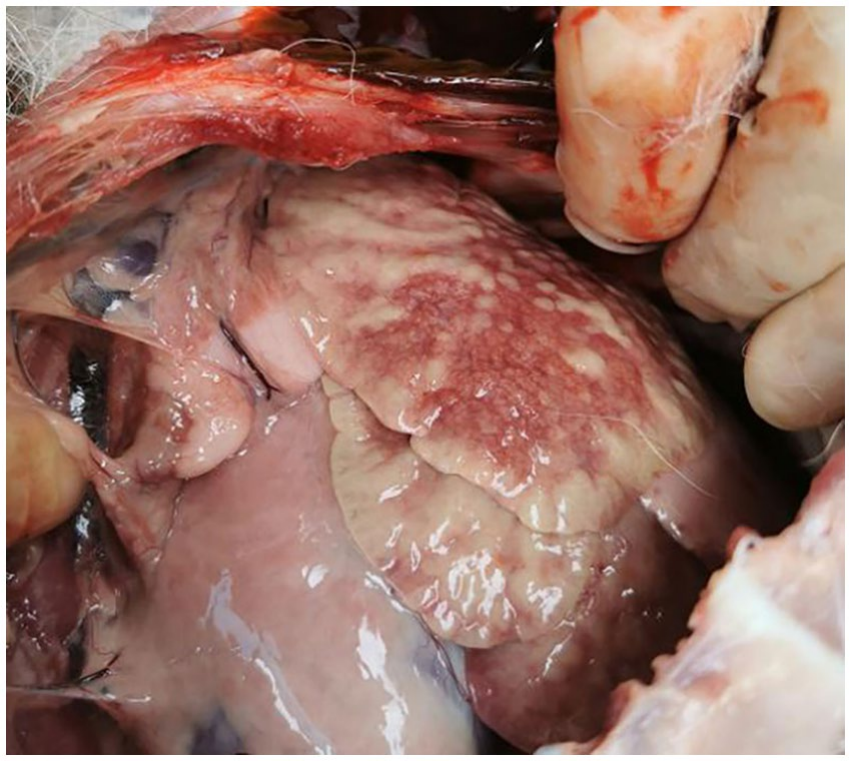
The lung lesions of the sick dog.

**Figure 2:**
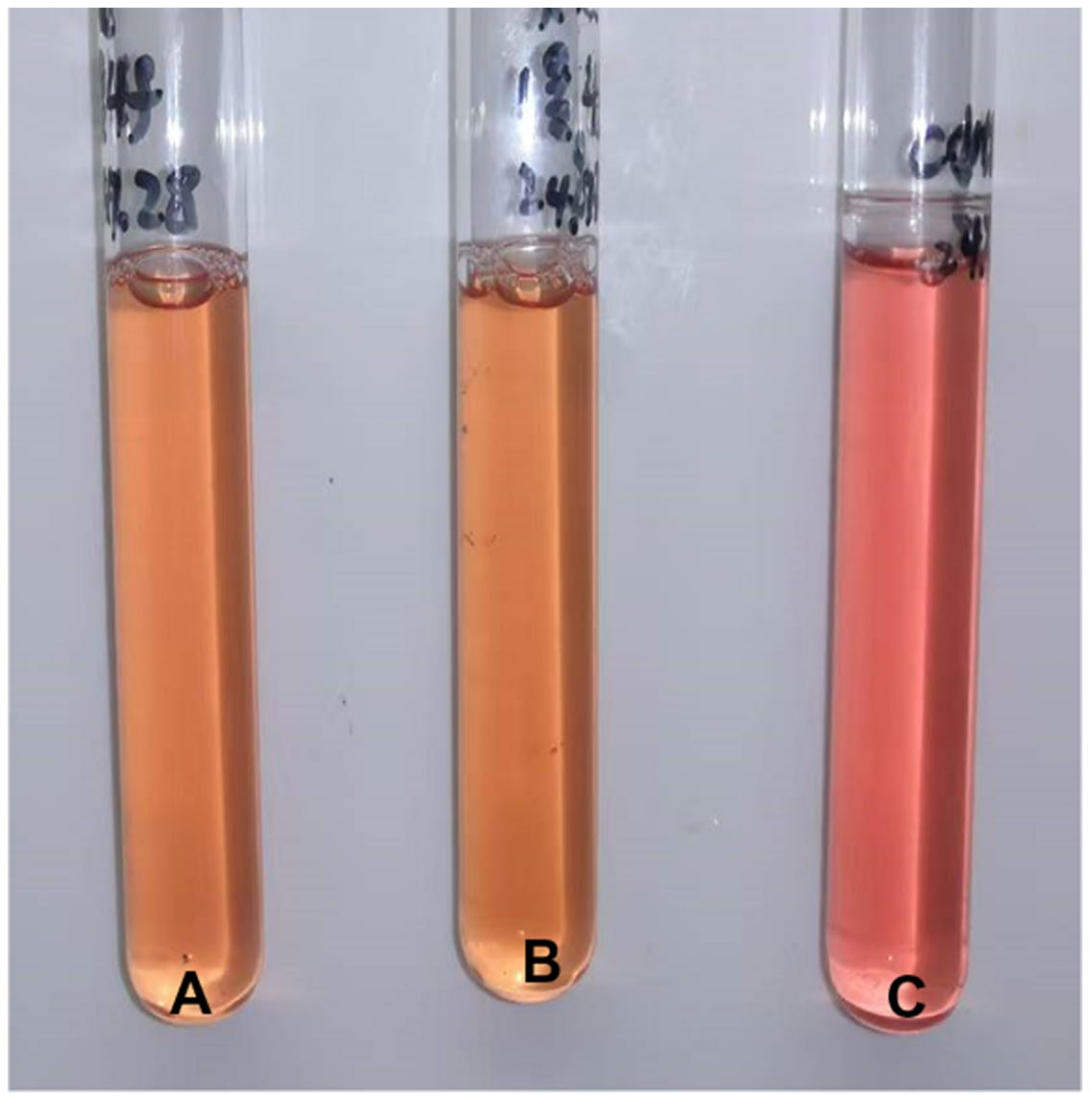
The color changes of *Mycoplasma canis* culture. **(A,B)** Yellow liquid tubes, both are mycoplasma culture tubes; **(C)** The red liquid tube is the negative control for PPLO liquid medium.

In the affected dogs, diffuse dark red consolidation areas were seen in the local lung lobes. The consolidation areas were mottled, and the boundary between the consolidation areas and normal lung tissue was unclear, showing a “map-like” fusion trend, which was consistent with hemorrhagic inflammatory changes. Scattered radial bright red congestion bands with a width greater than 3 mm suggest inflammatory congestion. Enhanced reflection on the surface of lung tissue suggests interstitial edema or alveolar edema. Local changes in the texture of lung tissue and loss of normal elasticity suggest lung consolidation. The color of non-consolidated lung tissue is relatively normal, but it appears slightly pale due to compensatory emphysema. The pleural layer was intact and smooth, with no fibrinous exudation observed (Figure 1). Tissue blocks were collected from areas with obvious lesions and then cut and ground. After liquid filtration, PPLO liquid medium was inoculated for mycoplasma culture. Three days later, one-tenth of the volume was filtered through a 0.45 nm filter membrane and continued to be subcultured. It could be seen that the medium showed a significant color reaction, and the liquid in the culture tube for mycoplasma growth was yellow or orange. The control tube’s liquid remains red (Figure 2).

### 3.2 16S rRNA sequence analysis

**Figure 3:**
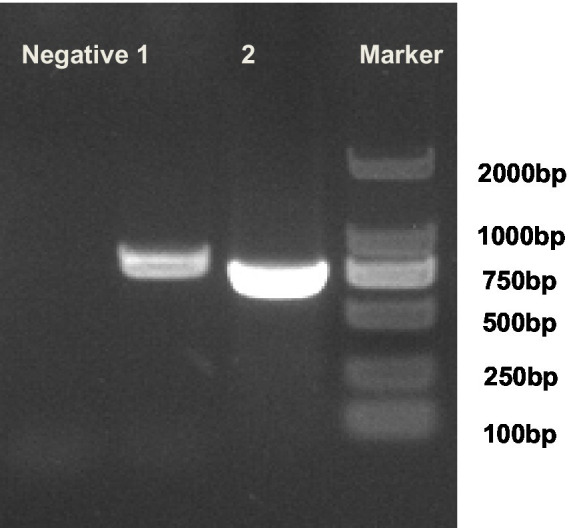
Test results of *M. canis* 16S rRNA PCR.1: *M. canis* 16S gene Seg-2 fragment, *M. canis* 16S gene Seg-1 fragment. 2: The positive plasmid synthesized from the *M. canis* 16S rRNA gene was used as the positive control. The negative control was nuclease-free sterile water.

**Figure 4:**
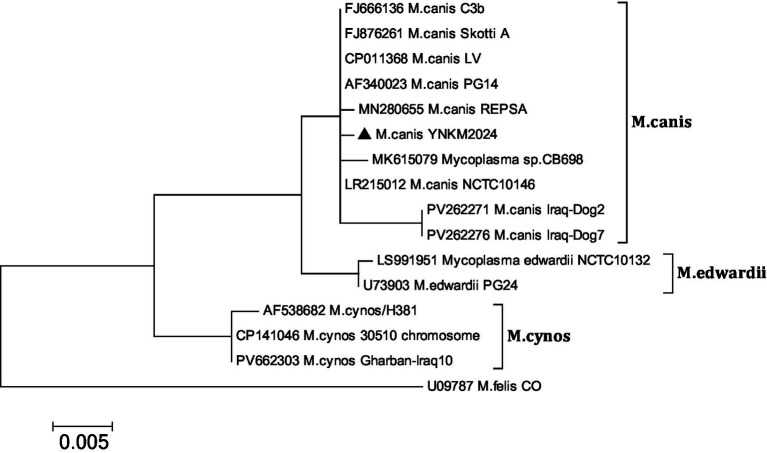
Phylogenetic analysis was conducted on the 16S RNA gene of *M. canis* isolated by us, including the nucleotide sequence distances of the corresponding reference strain genes. The black triangles represent the isolated strains in this study. The nucleotide sequences were aligned by the Clustal W method. The phylogenetic tree was constructed using MEGA 6.0 software based on the Kimura-2 parametric model to evaluate the evolutionary relationships among different strains. The adjacency algorithm is adopted to conduct 1,000 bootstrap repetition tests. A bootstrap value (%) ≥ 50% is displayed at the tree branch node. The scaly scale indicates the length of the branch.

The 16S gene fragments detected in two segments were all successfully amplified by electrophoresis verification (Figure 3). The two 16S gene sequences were combined using SeqMan software to obtain the 16S near-full-length gene fragment. The results of gene sequence analysis showed that the homology with the NCTC10146 strain in the UK in 1951 was 99.86%, the homology with the PG14 strain in the US in 2001 and the LV strain in 1992 was 99.79%, and the homology with the IRAQ-DOG2 and dog7 strains that were prevalent in Iraq in 2025 was both 97.98%. The homology with the Edward genotype (M. Edward II strain) PG24 strain and NCTC10132 strain was 98.9 and 98.83% respectively, and the homology with the *M. cynos* genotype H381 strain published by the UK in 2002 was 97.58%. The mycoplasma isolated this time can be classified to the *M. canis* genotype based on 16S rRNA (Figure 4).

### 3.3 Growth curve

**Figure 5:**
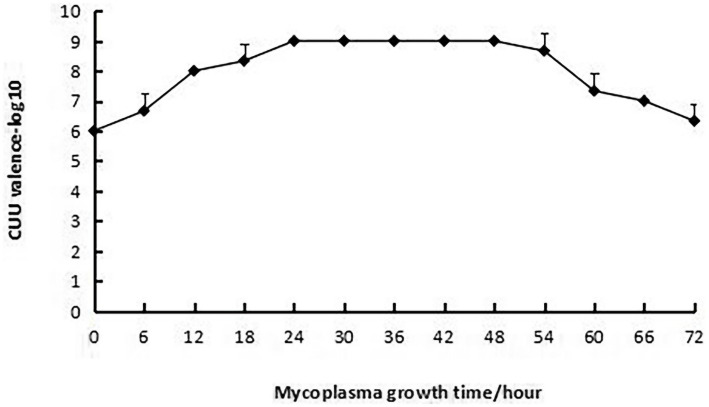
The growth curve of *M. canis* in PPLO medium. Mycoplasma in dogs begins to reproduce within 6 h after inoculation. The growth of mycoplasma reaches its peak at 24 h, and the peak can be stably maintained until the 48th h at 37 °C.

Freshly cultured *M. canis* was used as the strain and inoculated into PPLO liquid medium at a rate of 1% (V/V). Samples were taken every 6 h and CCU detection was conducted. The results showed that mycoplasma began to reproduce within 6 h after inoculation. The growth of mycoplasma reached its peak at 24 h, at 10 ^ 9 CCU/0.1 mL. The peak could be stably maintained until the 48th hour at 37 degrees, after which the number of live mycoplasma began to decline. The negative exponent of the three repeated data at each time point ^−^x ± SD is between 0 and 0.577, and the coefficient of dispersion is between 0 and 9.17%, so the result is reliable (Figure 5).

### 3.4 The influence of pH value on the stability of *M. canis*

**Figure 6:**
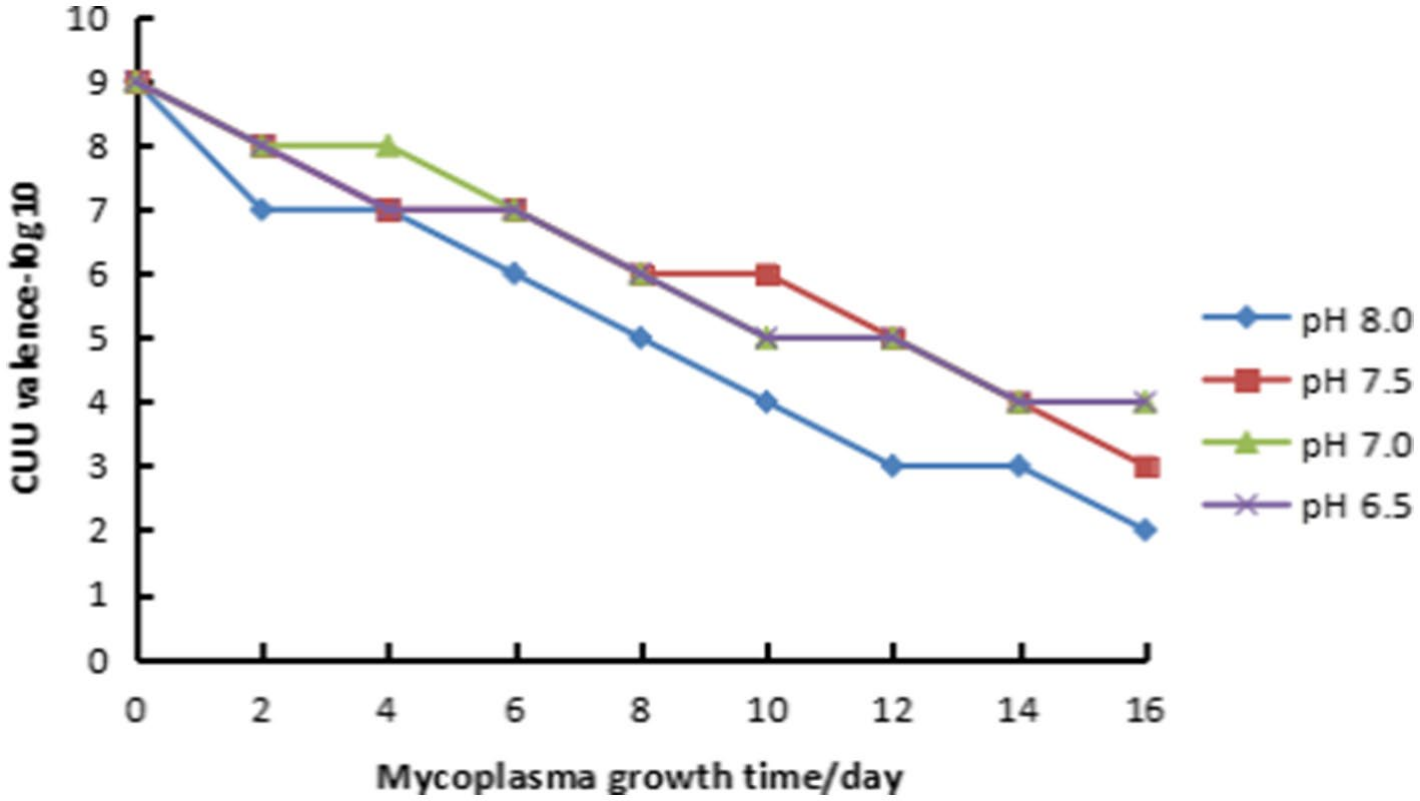
The trend of activity changes of the YNKM-2024 strain when stored at 4 °C under different pH values. The *M. canis* YNKM-2024 strains with pH values of 6.5, 7.0, 7.5, and 8.0, respectively, in the PLO medium showed a significant decreasing trend in the number of viable mycoplasma under the storage condition of 4 °C.

The test results showed that the *M. canis* YNKM-2024 strains with pH values of 6.5, 7.0, 7.5, and 8.0 in the PPLO medium all showed a significant decreasing trend in the number of viable mycoplasma under the storage condition of 4 °C (Figure 6). By the 16th day, they decreased to 10 ^ 4, 10 ^ 4, 10 ^ 3, and 10 ^ 2 CCU/0.1 mL, respectively. Overall, the loss rate of mycoplasma live at pH values of 6.5, 7.0, and 7.5 is lower than that at pH 8.0. Therefore, when the *M. canis* strain used as a species is stored at 4 °C, the pH value should be maintained at neutral or weakly acidic, and the storage time should be strictly limited.

### 3.5 The immunization results of inactivated vaccines

After being cultured in PPLO medium for 7 days with inactivated mycoplasma, no color change was observed in the CCU. It was determined that there was no mycoplasma propagation and the inactivation was complete. It can be used for the preparation of inactivated vaccines and animal experiments. The detection results of serum antibodies in the inactivated vaccine group and the control group based on emulsification with Montanide Del 02 PR adjuvant are shown in Table 3. The results showed that all four dogs vaccinated with inactivated vaccines produced antibodies against mycoplasma within 1 week after vaccination, with a PI value of ≥40%, while the control group that did not receive the inactivated mycoplasma vaccine was negative. This indicates that inactivated vaccination can enable dogs to produce antibodies rapidly. The calculation method of PI value is as follows: Inhibition rate (PI) = (1- sample OD value/negative control OD value) × 100%. And all the dogs performed normally after vaccination without any adverse reactions. This vaccine trial only detects antibody response, no data on protection, cytokines, or challenge test. And it is a preliminary immunogenicity assessment. Next steps challenge trials to confirm efficacy.

**Table 3:** Results of antibody detection in dogs after vaccination with inactivated *Mycoplasma canis* vaccine.

Group	Dog number	Vaccination time			
		0 w	1 w	2 w	3 w
Vaccination group	7,599	11.55%	54.13%	55.29%	55.28%
		12.23%	50.83%	54.88%	52.51%
	1,514	15.40%	53.33%	54.82%	60.64%
		10.52%	56.85%	58.69%	56.45%
	6,933	7.78%	56.11%	53.16%	60.53%
		7.74%	41.95%	54.93%	50.07%
	4,806	28.43%	46.51%	48.05%	52.72%
		21.34%	49.80%	46.48%	54.02%
Negative control group	7,124	1.36%	0.00%	23.12%	6.69%
		0.00%	0.00%	21.29%	9.04%
	8,812	17.11%	17.17%	8.61%	27.07%
		19.00%	17.73%	10.38%	25.78%

## 4 Discussion

### 4.1 Research overview of *M. canis*

Research literature on canine mycoplasma is relatively scarce, with only a dozen or so related papers published in the past 20 years. This may be related to the fact that dogs are not intensively farmed in most regions. Additionally, in most cases, existing antibacterial drugs can effectively inhibit or kill canine mycoplasma, so its pathogenicity has not received sufficient attention. However, with the widespread use of antibacterial drugs, the problem of drug resistance in mycoplasma has become increasingly serious in human *Mycoplasma pneumoniae*, *Mycoplasma bovis* and avian mycoplasma (7, 8), and canine mycoplasma is also facing this potential threat. Given the important and irreplaceable role of dogs as family pets and working dogs, it is currently urgent to strengthen research on canine mycoplasma, covering multiple aspects such as epidemiology, diagnostic techniques, drug resistance mechanisms and vaccine development.

The 16S rRNA gene is a highly conserved gene in prokaryotes, responsible for encoding part of the ribosomal RNA. By comparing the 16S rRNA gene sequences of different species, researchers can reveal their phylogenetic relationships (9, 10). In this study, after comparing and analyzing the 16S rRNA gene sequences of canine mycoplasma, it was found that the isolated canine mycoplasma was highly similar to the sequences of *M. canis*, *M. cynos* and *Mycoplasma edwardii*, with homology exceeding 97%. Therefore, based on the analysis of this gene, it may be difficult to achieve precise genotyping and can only be used as a preliminary classification basis. This result is consistent with the research conclusions of Victoria and Chalker (11). More in-depth classification and functional research still need to rely on more gene sequence information, gene function analysis and systematic exploration of the infection mechanism.

### 4.2 Research on the growth characteristics and vaccine characteristics of *M. canis*: providing insights for prevention and vaccine development

The *M. canis* isolated in this study showed good growth ability in PPLO medium. When inoculated with fresh culture at a 1% inoculation volume, the peak bacterial concentration was reached within 24 h, and a significant acidic reaction (turning yellow) was observed in the phenol red indicator. However, if frozen or stored at 4 °C for a period of time, it may take 36 h or even longer to observe the acidification of the culture medium. Therefore, in the process of monitoring mycoplasma growth, pH value changes should be used as the main basis for judging the growth state, rather than relying solely on culture time. The temporary preservation of mycoplasma strains at 4 °C is an important step in mycoplasma research and vaccine production. Although researchers know that mycoplasma is unstable and easily loses activity under normal or low-temperature storage conditions, their understanding of the degree of activity reduction is intuitive, especially under different pH conditions. This study’s quantitative detection and evaluation of the viability of *M. canis* under 4 °C storage conditions and different pH values provide important reference data for future research and application of *M. canis* and other mycoplasma.

Due to research conditions, the host return experiment of mycoplasma infection cannot be carried out for the time being, and it is currently impossible to prove the pathogenic role of *M. canis* in canine respiratory diseases, especially in the process of typical lobar pneumonia in the lungs. However, based on the detection results of dogs with obvious respiratory symptoms and the results of strain isolation, it can be inferred that when dogs have weakened resistance due to infection with other viral diseases or stress responses, it may play a major or synergistic pathogenic role.

Inactivation of mycoplasma has been successful in the research of other types of mycoplasma, and the adjuvant and delivery methods have been optimized, resulting in good protective effects (12–14). BEI, as an inactivating agent that can directly destroy the nucleic acid of viruses or mycoplasma, has been widely used (15, 16). This study successfully applied the BEI inactivator to inactivate *M. canis*, and the prepared vaccine induced antibodies in vaccinated dogs, indicating that this preparation method can maintain the good immunogenicity of *M. canis* and stimulate the humoral immune system of the body to produce specific antibodies. However, due to the limitations of conditions, no mycoplasma challenge test was conducted. The existing experiments cannot prove whether the produced antibodies have protective effects and the extent of such protection. As a preliminary study, at least it shows that this inactivation procedure and vaccine preparation are feasible, although it may not be the optimal choice. Challenge experiments, adjuvant screening and other studies will be carried out in subsequent research.

## 5 Conclusion

This study isolated and identified *M. canis*, studied its culture characteristics and stability on PPLO medium, and provided a method and preliminary evaluation for the preparation of *M. canis* inactivated vaccines. It provides a basis for further research on the infection characteristics and pathogenicity of *M. canis*, and provides a reference for the prevention and control of *M. canis* infection and transmission.

## Data Availability

The datasets presented in this study can be found in online repositories. The names of the repository/repositories and accession number(s) can be found in the article/supplementary material.
